# Expression Systems and Species Used for Transgenic Animal Bioreactors

**DOI:** 10.1155/2013/580463

**Published:** 2013-03-17

**Authors:** Yanli Wang, Sihai Zhao, Liang Bai, Jianglin Fan, Enqi Liu

**Affiliations:** ^1^Laboratory Animal Center, Xi'an Jiaotong University School of Medicine, Xi'an, Shaanxi 710061, China; ^2^Xi'an Jiaotong University Cardiovascular Research Center, Xi'an, Shaanxi 710061, China; ^3^Department of Molecular Pathology, Interdisciplinary Graduate School of Medicine and Engineering, University of Yamanashi, Yamanashi 409-3898, Japan

## Abstract

Transgenic animal bioreactors can produce therapeutic proteins with high value for pharmaceutical use. In this paper, we compared different systems capable of producing therapeutic proteins (bacteria, mammalian cells, transgenic plants, and transgenic animals) and found that transgenic animals were potentially ideal bioreactors for the synthesis of pharmaceutical protein complexes. Compared with other transgenic animal expression systems (egg white, blood, urine, seminal plasma, and silkworm cocoon), the mammary glands of transgenic animals have enormous potential. Compared with other mammalian species (pig, goat, sheep, and cow) that are currently being studied as bioreactors, rabbits offer many advantages: high fertility, easy generation of transgenic founders and offspring, insensitivity to prion diseases, relatively high milk production, and no transmission of severe diseases to humans. Noticeably, for a small- or medium-sized facility, the rabbit system is ideal to produce up to 50 kg of protein per year, considering both economical and hygienic aspects; rabbits are attractive candidates for the mammary-gland-specific expression of recombinant proteins. We also reviewed recombinant proteins that have been produced by targeted expression in the mammary glands of rabbits and discussed the limitations of transgenic animal bioreactors.

## 1. Introduction

The term bioreactor, which originally meant a tank in which cells, cell extracts, or enzymes carried out a biological reaction, now often refers to a growth chamber (fermenter or fermentation vessel) for cells or microorganisms used in the production of recombinant proteins. Transgenic animal bioreactors used to produce therapeutic proteins have existed for decades; several proteins produced in these systems are now in clinical trials, and one has been approved for marketing. 

Compared with other systems of production for recombinant proteins, transgenic animal bioreactors are, overall, an attractive platform because transgenic animal bioreactors represent powerful tools to address the growing need for therapeutic recombinant proteins. The ability of transgenic animals to produce complex, biologically active proteins in an efficient and economic manner is superior to those of bacteria, mammalian cells, transgenic plants, and insects [1]. As we know, bacteria are limited in their ability to perform the posttranslational protein modifications necessary for many targets [2, 3], and transgenic plant [4–6] and insect production systems involve relatively slow production set-ups and have yet to cross many regulatory hurdles [7–9]. A comparison of the different systems used to produce recombinant pharmaceutical proteins is summarized in Table 1. Another important consideration is cost. Although direct comparison of the production costs associated with these different systems is rather difficult, a previous study suggested that building a large-scale (10,000 liter bioreactor) manufacturing facility for mammalian cells takes 3–5 years and costs US$ 250–500 million, whereas a transgenic farm with a single purification facility should not cost more than US$ 80 million and would most likely cost less [7]. Establishing a commercial production herd of a company's transgenic goats could be accomplished at approximately a tenth of the cost of building a commercial cell-culture facility [10]. As shown in Table 2, protein production costs are substantially lower for transgenic animals than for cell culture. Therefore, transgenic animal bioreactors show a financial advantage over cell culture or other systems, even when all costs are taken into account. 

Recombinant proteins are produced from transgenic animal body fluids. Milk, egg white, blood, urine, seminal plasma, and silkworm cocoon from transgenic animals are candidate sources of recombinant proteins produced at an industrial scale. 

The technology for using the mammary glands of transgenic animals as primary bioreactors has been developed for large transgenic animals, such as cows, goats, sheep, and pigs. Instead, many laboratories and pharmaceutical companies have made efforts to produce a variety of valuable therapeutic proteins using transgenic rabbits. In this paper, we compared different systems and species of transgenic animal bioreactors. 

## 2. Protein Production Platforms Using Transgenic Animals

In 1985, Hammer and colleagues established the first transgenic livestock animals, including sheep, rabbits, and pigs, in an attempt to develop a way to produce recombinant proteins from these animals [11]. Since then, production of a number of recombinant proteins from transgenic animals has been reported. Many laboratories and pharmaceutical companies have made efforts to produce a variety of valuable therapeutic proteins from transgenic animals, such as cows, pigs, sheep, goats, and rabbits [7, 8, 12, 13].

Selection of a suitable method for expressing a recombinant protein is dependent on the characteristics and intended application of the recombinant protein [14]. Presently, milk is the most mature system for producing recombinant proteins from transgenic organisms. Blood, egg white, seminal plasma, silk gland, and urine are other theoretically possible systems. The advantages and disadvantages of different expression systems are shown in Table 3. 

### 2.1. Mammary Gland, the Best Bioreactor Available

The mammary gland has generally been considered the organ of choice to express valuable recombinant proteins in transgenic animal bioreactors because milk is easily collected in large volumes. Milk is currently the best available bioreactor. Foreign proteins are commonly reported to be produced in transgenic milk at rates of several grams per liter. Based on the assumption of average expression levels, daily milk volumes, and purification efficiencies, 5.400 cows would be needed to produce the 100.000 kg of human serum albumin that are required per year worldwide, 4.500 sheep would be required for the production of 5000 kg *α*-antitrypsin (*α*-AT), 100 goats for 100 kg of monoclonal antibodies (mAbs), 75 goats for 75 kg of antithrombin III, and two pigs to produce 2 kg of human clotting factor IX [15]. As a result, a great deal of effort has been made to produce transgenic bioreactors not only with the traditional “dairy” species, such as sheep, goats, and cows, but also with rabbits and pigs. 

A number of examples leave no doubt about the capacity of the mammary gland to synthesize, mature, and secrete foreign proteins. Apart from these successes, a certain number of failures in animal mammary gland systems have occurred for various reasons: (1) purely technical issues such as problems in the generation of transgenic mammals, (2) the need for more fundamental knowledge in areas such as protein maturation or secretion, and (3) the fact that certain bioactive proteins produced in milk can have adverse affects on an animal's health; this is particularly true when they are produced at high concentrations and the protein can be reabsorbed.

### 2.2. Blood and Egg: Alternative Recombinant Protein Secretion Medium

The mammary gland of a transgenic animal is the most popular protein bioreactor. However, there are alternative systems based on production of useful pharmaceutical proteins in blood and eggs [16]. Animal blood, which collects secretions from many tissues, may be used as a source of recombinant proteins. For example, human *α*-AT was obtained at a high level from the serum of transgenic rabbits [17], and human hemoglobin has been produced in a transgenic swine circulatory system. This protein seemed to have been matured appropriately for its functionality [18, 19]. In principle, the human component of the pigs' blood was intended to be used as a blood substitute, but similarities between the porcine and human blood components made isolation of the human hemoglobin arduous. Additionally, recombinant antibodies were found in the blood of transgenic goats, pigs, and rabbits [20, 21]. Blood is a less-than-ideal fluid for protein production because its harvest is invasive and bioactive proteins could affect an animal's health to the point of making the system impractical.

The use of transgenic eggs for large-scale production of recombinant proteins is another method being contemplated. Interest in this system is driven by the fact that a single hen can produce an impressive number of eggs (up to 330 eggs/year) and egg white naturally contains approximately 4 g of protein [22–24]. Transgenic chicken stably produced a human erythropoietin fusion protein not only in their serum and egg white but also in the egg yolk, as was expected [25]. However, the egg system has been hampered by the lack of an efficient transgenesis system in poultry [7].

### 2.3. Urine- or Seminal-Fluid-Specific Expression Systems

Urine is an abundant biological fluid already used to prepare proteins such as gonadotropins for pharmaceutical use. If it happens that a foreign protein is matured in a more appropriate manner in the urothelium than in the mammary gland, or if the resulting side effects of the protein are less deleterious for the animals, a system using expression in the urothelium may be useful [26]. Work has indicated that expression of the human growth hormone gene driven by the mouse uroplakin II gene promoter was expressed specifically in the urothelium, and up to 100–500 ng/mL of human growth hormone was found in the resulting urine [27]. Other work has also been explored [28, 29]. Compared with milk, one advantage of using the bladder as a bioreactor is that animals can urinate earlier than they can lactate. The limiting factor for bladder production of proteins has been yield. Although the bladder epithelium does secrete proteins, the rates are minimal, and thus protein production rates with this system are extremely low.

The seminal fluid of the male ejaculate has also been considered as a site for recombinant protein secretion in transgenic animals [30]. Seminal fluid is a relatively abundant biological fluid in some species and it can be easily collected. This is the case for pig. The boar's male accessory sex glands possess many characteristics that make them appropriate for the production of recombinant proteins. Pig semen contains 30 mg of protein per mL and boars can produce 200–300 mL of semen for a total of 6–9 g of protein per ejaculate [7]. The collection and handling of boar semen is a well-established process, performed on a large scale at swine artificial insemination units worldwide. Also of interest is the fact that protein secretion by these tissues is uniquely exocrine, minimizing the risk of a biologically active recombinant protein upsetting the host's own physiology. The limitation of this system is that we do not know how complex proteins are matured and secreted in semen [26].

### 2.4. Silkworm Cocoon, a Good Candidate

The silkworm has acquired the ability to synthesize bulk amounts of silk proteins in its silk glands. To utilize this capacity for mass production of useful proteins, transgenic silkworms have been generated that synthesize recombinant proteins in the silk gland and secrete them into the silk cocoon; many recombinant proteins have been produced using this system, particularly over the last decade [31–33]. The transgenic silkworm is not only suitable for the production of genetically modified scaffolds for fibrous proteins such as collagens, elastin, and silk which can be used to produce fabrics and biomaterials for medical purposes, but also suitable for the production of recombinant proteins that can be used for pharmaceutical purposes. More specifically can yield up to 4 mg of recombinant protein per silkworm. This yield is quite high compared with other systems, and production using transgenic silkworms is much cheaper and faster than production using transgenic livestock [34]. The advantages of transgenic silkworms are convenience and cost effectiveness with increased product yields in most cases ranging from 20- to 10 000-fold compared with laboratory methods. The disadvantages are that it is costly and time consuming to maintain nondiapause transgenic silkworms and that the use of denaturing chemicals to extract the recombinant proteins may result in extracted proteins that cannot retain their original structures. 

### 2.5. Others

Fish may present other specific, unique, and unexplored opportunities for use of transgenic animals as bioreactors for production of important proteins. Some biopharmaceutical companies have ongoing projects to express factor VII, insulin, collagen, human calcitonin, pleurocidin, and human defensins in mucus produced by tilapia and salmon [35]. However, at present, we have little knowledge about transgenic fish bioreactors. 

The use of transgenic plants to produce novel products has great biotechnological potential, as they are relatively inexpensive, safe sources for potentially valuable bioactive metabolites, diagnostic proteins, and vaccines [4–6]. For example, nicotiana hybrids provide an advantageous production platform for partially purified, plant-made vaccines that may be particularly well suited for use in veterinary immunization programs [36]. Transgenic plants are superior in terms of storage and distribution issues.

## 3. Recent Successes: Different Animals Used as Mammary Gland Bioreactors

As what we discussed in Section 2.1, transgenic animal mammary glands are the best available bioreactors. They can express a variety of interesting recombinant proteins—large and small, simple and complex—with high efficiency and full bioactivity and have been extensively and successfully used in different animals.

Transgenic mice may only serve as a predictive model to evaluate the usefulness of expression constructs and to study the properties of expressed proteins. However, they are not at present useful as bioreactors for producing the large quantities of recombinant proteins that can satisfy commercial demands. 

The largest and most complex protein successfully produced to date is the human clotting protein factor VIII, a large heterodimer that was correctly processed into a bioactive protein in pigs [67]. The gene contains 26 exons and the cDNA alone is 7.6 kb long. At the other extreme, small peptides are unstable in biological systems, but they can be expressed in transgenic animals if fused to carrier proteins. For example, the salmon calcitonin peptide was fused to a small milk protein (human a-lactalbumin) and successfully expressed and amidated in rabbits [61]. Calcitonin was cleaved from the fusion precursor in vitro during the purification process to yield a peptide with potent bioactivity in vivo. This is one approach to expressing physiologically active polypeptides without compromising the physiology or health of the transgenic production animal. Even multimeric proteins have been expressed and assembled in vivo by the coinjection of separate transgene constructs containing the individual protein chains. Correct assembly into bioactive proteins requires coexpression of the individual proteins at the same time in the same cell with the correct stoichiometry. This has been accomplished in vivo for heterodimeric mAbs expressed in mice and goats and for heterotrimeric fibrinogen produced in sheep.

Proteins that require posttranslational modification (e.g., glycosylation) have been expressed successfully, including anti-thrombin-III in goats [68] and extracellular superoxide dismutase, a complex N-glycosylated homotetramer that carries copper and zinc atoms and is sensitive to proteolysis, produced in physiologically active form in transgenic rabbits [47]. In recent years, several bioactive proteins have been expressed successfully in different animal mammary glands [69], such as lactoferrin [70, 71], human parathyroid hormone [72], *α*-fetoprotein [73], lysozyme [74], and butyrylcholinesterase [75]. 

ATryn, produced by GTC Biotherapeutics, is made from the milk of transgenic goats that produce human antithrombin, a plasma protein with anticoagulant properties [76]. Early in 2006, the European Medicines Agency approved ATryn for use in European Union countries; the drug was also approved by the FDA in 2009 for treatment of patients with hereditary antithrombin deficiency. It was stated that one transgenic goat could produce the same amount of antithrombin in a year as 90.000 blood donations [77]. ATryn is the first medicine produced using transgenic animals. Its value is in the proof and acceptance of an intransgenic-animal platform to produce therapeutic proteins. Undoubtedly, an increasing number of drugs produced by transgenic animal mammary glands will be approved in the near future.

## 4. Rabbits versus Livestock: Bigger Is Not Always Better

Several animal species have been successfully used as transgenic bioreactors. However, the criteria for selecting the most suitable animal species for molecular pharming are based on the quantity of proteins needed per year, the capacity of a facility, and the potential commercial value of the recombinant proteins in addition to other factors such as time until milk production and milk volume. The features of milk secretion in livestock are summarized in Table 4. A simplified rule for choosing transgenic bioreactors is that the production of a protein (such as albumin) in tons should be carried out using transgenic cows, in hundreds of kilograms using sheep or goats, and in kilograms per year using rabbits [78]. For example, in goats, a lactating female can produce up to 600–800 L of milk per year that can contain approximately 5 g/L recombinant protein to yield approximately 4 kg of protein per year.

In comparison with other large domestic livestock species, the rabbit is a relatively small animal with a short gestation time, sexual maturity period (only four months for females and five months for males) (Table 5), and optimal size. Handling in reproduction favors the application of transgenic technology in rabbits; gene transfer into rabbits is an attractive technique for improving their performance, and applications have been developed that use rabbits as fast bioreactors for the production of therapeutic proteins used in biomedical research [78]. Rabbits are efficient breeders and will produce milk containing a desired protein within 8 months after the start of a project (Table 4). 

Rabbit milk naturally contains 2.5-fold as much protein as sheep milk and 4.8-fold that of goat milk. A lactating female rabbit can produce 170–220 g of milk per day and yield up to 10 kg of milk per year under semiautomatic hygienic milking conditions [79]. Expression levels of transgenic protein can be as high as 20 grams per liter. For small- and medium-sized facilities, the rabbit system is ideal to produce up to 50 kg of protein per year. Thus, the transgenic rabbit system is a lower cost alternative primarily because rabbits are smaller and less expensive to maintain than livestock. 

Specific pathogen-free rabbits are available and free of infectious agents. Although for conventional rabbits there are no known prion diseases (similar to scrapie of the sheep, human Creutzfeldt-Jacob disease and kuru, bovine spongiform encephalopathy of the bovines) [80], humans can be infected with avian influenza, a virus from poultry [81], though rabbit. There has been no known serious disease transmission to humans from the rabbit, which makes the rabbit safer than other dairy livestock and poultry. Therefore, the system of transgenic rabbits is safe to produce therapeutic proteins. 

## 5. Transgenic Rabbit Mammary Gland Bioreactor

Considering both economical and hygienic aspects, rabbits are attractive for the mammary-gland-specific expression of recombinant proteins. Using an appropriate promoter, a number of recombinant proteins have been produced from rabbit milk, including hormones, bioactive peptides, and therapeutic proteins. Recombinant human proteins produced by transgenic rabbits include *α*-AT [17], interleukin-2 [51], tissue plasminogen activator [57], erythropoietin [42–46], insulin-like growth factor-1 [52–54], extracellular superoxide dismutase [47], growth hormone [48–50], *α*-glucosidase [37, 38], salmon calcitonin [61], equine chorionic gonadotropin [60], nerve growth factor-*β* [55, 56], chymosin [58], C1 inhibitor [39], clotting factor VIII [40, 41], tissue nonspecific alkaline phosphatase [63], bovine follicle-stimulating hormone [59], protein C [62], lactoferrin [64], interferon beta [65], and antithrombin [66], as summarized in Table 6.

One of the best examples of recombinant proteins reported from rabbit milk is human *α*-glucosidase, which was the first transgenic product from rabbit milk used to treat Pompe's disease (also called glycogen storage disorder type II) [37, 82, 83]. Pompe's disease is a fatal muscular disorder caused by lysosomal *α*-glucosidase deficiency; patients with this disease have a rapidly fatal or slowly progressive impairment of muscle functions due to concomitant storage of lysosomal glycogen in the muscles and massive cardiomegaly. In 1998, a group of scientists in The Netherlands generated transgenic rabbits using a fusion between the human acid *α*-glucosidase gene in its genomic context and the bovine *α*-S1-casein promoter. This protein isolated from transgenic rabbit milk was shown to exert therapeutic effects in the treatment of mice with glycogen storage deficiency and later in the treatment of human *α*-glucosidase deficiency [37]. Subsequently, they administered recombinant human *α*-glucosidase from rabbit milk to four human babies who were genetically deficient in *α*-glucosidase, at starting doses of 15 or 20 mg/kg and later at 40 mg/kg. The activity of human *α*-glucosidase was shown to be normalized in the muscles of these patients, and their tissue morphology and motor and cardiac functions were dramatically improved [82]. That successful study provided convincing evidence that the milk of transgenic rabbits is a safe source of therapeutic proteins and has opened the way for further exploration of this production method. 

It must be admitted that not all transgenic rabbit bioreactors or the recombinant proteins they produce are functional or practical due to low levels of expression; however, these studies have opened the door for possible technical advances that will permit the production of large quantities of human therapeutic proteins and their use in the future. 

## 6. Conclusions

The various mammals used as bioreactors are rabbits, pigs, sheep, goats, and cows. Each of these species offers advantages and drawbacks. Rabbits are sufficient to produce several kilograms of proteins per year. The rabbit is particularly flexible, allowing rapid generation of founders and scaling-up. For very high protein production, larger animals are needed. The recombinant proteins that have been prepared in milk are mainly naturally secreted, which may facilitate or complicate their purification. No matter what type of transgenic platform is used as a bioreactor, guidelines developed by the FDA require monitoring of the animals' health, validation of the gene construct, and characterization of the isolated recombinant protein as well as the performance of the transgenic animals over several generations. This has been taken into account in the development of gene pharming, for example, by using only animals from prion disease-free countries (New Zealand) and keeping the animals in very hygienic conditions. It may thus be considered that the preparation of pharmaceutical proteins from milk is a safe process.

The raw potential for producing valuable proteins with transgenic animals seems apparent. The study of the properties of the recombinant proteins is of paramount importance before they can be put onto the market. Although this problem is particularly complex because natural and recombinant proteins often exist in different forms, the successful drug ATryn from transgenic goat approved by the European Union and the FDA will demonstrate the usefulness and solidity of this approach and will accelerate registration of further products from this process as well as stimulate research and commercial activity in this area. 

The transgenic production of recombinant proteins offers a safe, efficient, and economical way to manufacture valuable biotherapeutics. A number of recombinant proteins have been produced in several transgenic animal species. However, current methods of generating transgenic animal founders are relatively inefficient and time consuming, and attempts to improve transgenesis by various methods have had limited success. The inefficiency of transgenesis in dairy species, as well as certain innate disadvantages of lactation, has prompted interest in expressing foreign proteins in various tissues of more prolific species. In addition, the purification of recombinant proteins from milk is still a hurdle to be overcome and creates often undefined regulatory issues. 

## Figures and Tables

**Table 1:** Comparison of the different systems used to produce recombinant pharmaceutical proteins.

	Bacteria	Mammalian cells	Transgenic animals
Production level	++	+	++++
Investment cost	+++++	+	+++
Production cost	+++++	++	++++
Scaling-up ability	+++++	+	++++
Collection	+++++	+++++	++++
Purification	+++	++++	+++
Posttranslational modifications	+	++++	++++
Glycosylation	+	++++	++++
Stability of product	+++++	+++	++++
Contaminant pathogens	+++++	++++	++++
Products on the market	++++	+++++	+++

Table adapted from [1].

**Table 2:** Comparative estimated production cost between cell culture and transgenics.

Production scale (Kg/year)	System	Cost (dollars/gram product)
50	Cell culture	147
	Transgenics	20
100	Cell culture	48
	Transgenics	6

Table adapted from [7].

**Table 3:** Comparison of the different transgenic animal species used to produce recombinant pharmaceutical proteins.

Points to consider	Production systems						
	Milk	Blood	Egg white	Seminal fluid	Urine	Silk cocoon	Others
Production level	+++++	+++++	+++++	+++	++	++	++
Investment cost	+++	+++	+++	+	+	+++	+++
Production cost	++++	++++	++++	++	+	+++++	++++
Scaling-up	++++	++++	++++	++	+	++++	+++
Collection	+++++	++++	+++++	+++	+++	+++++	+++++
Purification	+++	++	+++	++	++	+++	++
Effect on organism	+++	++	+++	+++	+++	++++	++++
Posttranslational modifications	++++	+++++	+++	+++	+++	++	++
Glycosylation	++++	++++	+++	+++	+++	++	++
Contaminant pathogens	+++	++	+++	+++	++	++++	++++
Products on the market	++++	+	++	+	+	++	+

Table adapted from [1].

**Table 4:** Comparison of transgenic milk expression systems in different species.

Species	Gestation (months)	Maturation (months)	Milk yield per lactation (L)	Elapsed months from microinjection to milk
Mouse	0.75	1	0.0015	3–6
Rabbit	1	5-6	1–1.5	7-8
Pig	4	7-8	200–400	15-16
Sheep	5	6–8	200–400	16–18
Goat	5	6–8	600–800	16–18
Cow	9	15	6000–8000	30–33

**Table 5:** Reproductive performance of rabbits.

Reproductive parameter	Value
Sexual maturity	4-5 months
Conception rate	65%
Gestation time	30–33 days
Litter size	5–12
Lactation period	40–50 days
Litter interval	44 days
Litters per year	4–7

**Table 6:** Recombinant proteins produced from transgenic rabbits.

Expressed proteins	Promoter	Expressed protein	References
Human *α*1-antitrypsin	Human *α* 1-antitrypsin DNA	1 g/L in plasma	[17]
Human *α*-glucosidase	Bovine *α*s1-casein	8 g/L	[37]
	N-acetyl-*β*-glucosaminyl	NA	[38]
Human C1 inhibitor	NA	NA	[39]
Human clotting factor VIII	Mouse WAP	NA	[40]
	Mouse WAP	0.005–0.161 g/L	[41]
Human erythropoietin	Rabbit WAP	0.0000003 g/L	[42]
	Rabbit WAP	NA	[43]
	Bovine *β*-lactoglobulin	0.5 g/L	[44]
	Rabbit WAP	NA	[45]
	Rabbit WAP	60–178 IU/L	[46]
Human extracellular SOD	Mouse WAP	3 g/L	[47]
Human growth hormone	Mouse WAP	0.000012 g/L	[48]
	Rat WAP	0.5–1.0 g/L	[49]
	Rat WAP	0.010 g/L	[50]
Human IL-2	Rabbit *β*-casein	0.0005 g/L	[51]
Human insulin-like growth factor	Bovine *α*s1-casein	1 g/L	[52]
	Bovine *α*s1-casein	0.3 g/L	[53]
	Bovine *α*s1-casein	0.678 g/L	[54]
Human nerve growth factor *β*	Bovine *α*s1-casein	0.25 g/L	[55]
	Adenoviral	0.346 g/L	[56]
Human tPA	Bovine *α*s1-casein	0.00005 g/L	[57]
Bovine chymosin	Bovine *α*s1-casein	1.5 g/L	[58]
Bovine FSH	Bovine *α*s1-casein	0.1 g/L	[59]
Equine chorionic gonadotropin	Rabbit WAP	0.022 g/L	[60]
Salmon calcitonin	Ovine *β*-lactoglobulin	2.1 g/L	[61]
Human protein C	Mouse WAP	0.0000001–0.0000003 g/L	[62]
TNAP	Human WAP	NA	[63]
Human lactoferrin	Adenoviral	2.3 g/L	[64]
Human interferon beta	NA	2.2–7.2 × 107 IU/L	[65]
Human antithrombin	Adenoviral	4.8 g/L	[66]

FSH: follicle stimulating hormone; IL-2: interleukin-2; NA: not available; SOD: superoxide dismutase; TNAP: tissue-nonspecific alkaline phosphatase; tPA: tissue plasminogen activator; WAP: whey acidic protein.
